# Improving Clinical Care in Tobacco and Smoking-Related Problems: A Report of Clinical Audit and Quality Improvement Project

**DOI:** 10.1192/bjo.2022.424

**Published:** 2022-06-20

**Authors:** Jiann Lin Loo, Jawad Raja, Ugochukwu Anyanwu, Nikhil Gauri Shankar, Asmaa Elsayed, Zeenish Azahr, John Clifford, Faye Graver

**Affiliations:** Betsi Cadwaladr University Health Board, Wrexham, United Kingdom

## Abstract

**Aims:**

Around 40% of people with serious mental health problems smoke, which is significantly higher compared to the general population of the United Kingdom. The Welsh Government has set the target to reduce the overall prevalence of smoking in Wales to 16% from 19. In order to reduce the impact of smoking on the population, the first step is to identify the problem. Hence, a comprehensive history of smoking will help to identify the addiction-related problems. Hence, this combined clinical audit and quality improvement project (QIP) is aimed at the evaluation of the admission clerking around the assessment and management of smoking-related problems in an inpatient mental health unit.

**Methods:**

This clinical audit was carried out at the local inpatient general adult mental health units in Wrexham. It was based on NICE smoking guidelines “Smoking: acute, maternity and mental health services”. Clinically relevant information without personal identification information was collected based on a proforma. The first re-audit was repeated without a specific intervention to see any change in pattern and the need for intervention. This was followed by the first intervention, i.e., the sharing of a PowerPointTM presentation discussing commonly utilised measurement tools in the assessment of smoking-related behaviours and the second re-audit.

**Results:**

The first round of clinical audit involves 32 admissions, the first re-audit was 19 admissions, and the second re-audit was 37 admissions. The baseline showed 71.88% of inpatient admissions were asked about their smoking history, but only less than 10% of them were assessed in detail around the types and quantity of tobacco products, features of dependence and withdrawal, the motivation of the clients to quit smoking, and any help offered to the patients. The number of inpatient admissions which was assessed for their smoking-related behaviour dropped to 36.84% during the first re-audit, and less than 16% of them were assessed in detail. The number improved slightly to 57.14% after the first intervention, although less than 40% of the inpatient admissions were assessed in detail.

**Conclusion:**

There is an inconsistent pattern of change in the percentage, and it seems that the intervention leads to minimal improvement of the assessment of smoking-related problems during admission clerking. The minimal change may be attributable to the change in posting around the intervention period. The future plan includes a more regular intervention arranged around the beginning of new postings for doctors to ensure they have adequate exposure to the assessment of smoking-related addiction problems.

